# Teratoma of the mediastinum: a case report

**DOI:** 10.1186/1752-1947-5-193

**Published:** 2011-05-20

**Authors:** Ricardo D Vieira, Henrique Grimberg, Kiyomi K Uezumi, Lea MM Demarchi, Jeane M Tsutsui, Neuza HM Lopes, Whady Hueb

**Affiliations:** 1 Department of Atherosclerosis, Heart Institute of the University of São Paulo, Av Dr Eneas de Carvalho Aguiar 44, AB, Sala 114, Cerqueira César, São Paulo-SP, 05403-000, Brazil; 2 Department of Cardiology, Heart Institute of the University of São Paulo, Av Dr Eneas de Carvalho Aguiar 44, AB, Sala 114, Cerqueira César, São Paulo-SP, 05403-000, Brazil; 3 Department of Radiology, Heart Institute of the University of São Paulo, Av Dr Eneas de Carvalho Aguiar 44, AB, Sala 114, Cerqueira César, São Paulo-SP, 05403-000, Brazil; 4 Department of Pathology, Heart Institute of the University of São Paulo, Av Dr Eneas de Carvalho Aguiar 44, AB, Sala 114, Cerqueira César, São Paulo-SP, 05403-000, Brazil; 5 Department of Echocardiology, Heart Institute of the University of São Paulo, Av Dr Eneas de Carvalho Aguiar 44, AB, Sala 114, Cerqueira César, São Paulo-SP, 05403-000, Brazil

## Abstract

**Introduction:**

This case report illustrates a rare case of teratoma of the mediastinum which was continuous to the pericardium and caused extrinsic compression to the right atrium.

**Case presentation:**

A 22-year-old Caucasian man with no complaints or comorbid conditions presented to our hospital with obliteration of the right cardiophrenic sinus by a mass. A non-invasive investigation demonstrated a tumoral mass which was continuous to the pericardium and caused extrinsic compression to the right atrium. The clinical suspicion was a pericardial or bronchogenic cyst. Surgical and anatomopathologic findings led to the diagnosis of a mature cystic teratoma with atrophic thymic tissue at the external teratoma surface.

**Conclusion:**

We present an original report of a mature teratoma causing obliteration of the right cardiophrenic sinus with extrinsic heart compression. The diagnosis of this tumor is very difficult through non-invasive investigation.

## Introduction

A teratoma of the mediastinum is an uncommon germ cell tumor, principally when heart structures are involved [1]. Five percent of germ cell tumors are extragonadally located, and men are affected more than women [2]. Most mediastinal teratomas produce no symptoms, and they are more commonly associated with compression of adjacent structures, predominantly those of the respiratory system. Another signal is bleeding or rupture of the tumor into the bronchial tree, pleura, or pericardium. Digestive enzymes from pancreatic tissue or intestinal mucosa into the tumor produce this phenomenon. A rare finding associated with rupture is hair or sebaceous material expectoration [2,3]. The most common tumors found in the anterior mediastinum are of thymic, thyroid, or lymphoid origin and of pericardium or bronchogenic cyst or fat pad [4,5].

## Case presentation

**Figure 1:**
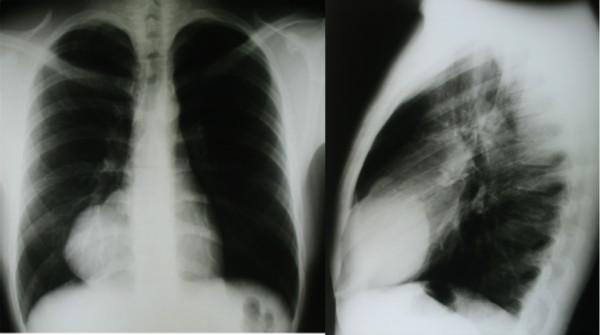
**Chest X-ray**.

**Figure 2:**
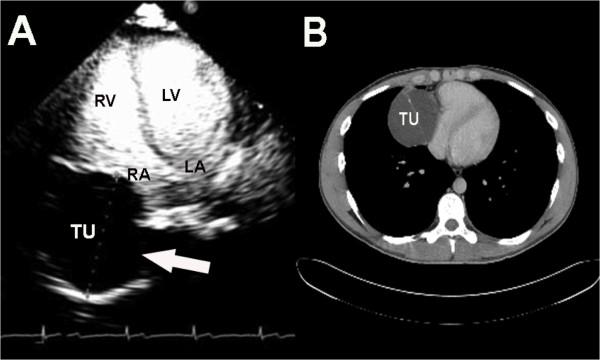
**Contrast echocardiographic and computed tomographic images**. **(A) **Contrast-enhanced echocardiographic image obtained after intravenous injection of lipid-encapsulated microbubbles. The apical four-chamber view demonstrates normal left ventricular opacification and low opacification of the tumoral mass. LA, left atrium; LV, left ventricle; RA, right atrium; TU, tumoral mass. **(B) **Computed tomographic image demonstrating a septate cystic mass containing septal calcification, which was continuous to the pericardium and caused extrinsic compression of the right atrium.

A 22-year-old Caucasian man with no complaints or co-morbid conditions presented to our hospital after an abnormal medical check-up before being hired for a job. A chest X-ray revealed an obliteration of the right cardiophrenic sinus by a bosselated mass widening the cardiac shadow (Figure 1). The young man was healthy and had good functional capacity. He denied fever, weight loss, previous disease, or any neoplasm history in his family. There was no previous chest X-ray. His physical examination revealed that he was apyrexial, normotensive, and eupneic. His thyroid, lymph node, chest, abdominal, and testis examinations were normal. A 12-lead electrocardiogram demonstrated sinus bradycardia. His chest X-ray showed a mass in the right cardiophrenic sinus with homogeneous, hazy density and a partially well-delineated margin continuous to cardiac shadow. A multi-detector computed tomographic scan showed a septate cystic mass containing septal calcification measuring 8.1cm×6.4cm, which was continuous to the pericardium and caused extrinsic compression of the right atrium. The mediastinal structures did not show any abnormal lymph nodes or features of compression or infiltration (Figure 2B). By this time, the suspected diagnosis was a pericardial cyst or fat pad. Magnetic resonance imaging suggested a cyst of the pericardium, but with heterogeneous cyst content. Transthoracic two-dimensional and real-time, live three-dimensional echocardiography revealed a normal-sized heart with normal function and blood flow velocities. A rounded extracardiac mass projecting to the right atrium was detected in the parasternal transverse view and apical four-chamber view. For further assessment of the suspicious mass, contrast-enhanced echocardiography was performed, which showed the clear definition of a rounded structure with low opacification with contrast (Figure 2A). Because of the mass feature, a benign tumor, probably a bronchiogenic cyst, was suspected.

Surgical excision was accomplished via a median sternotomy. This surgical access was chosen because of clinical suspicion that superior vena cava invasion would necessitate extracorporeal circulation. The tumoral mass was continuous to the right parietal pleura and pericardium. There was no cardiac, pulmonary, or vascular invasion. The surgical approach was successful.

**Figure 3:**
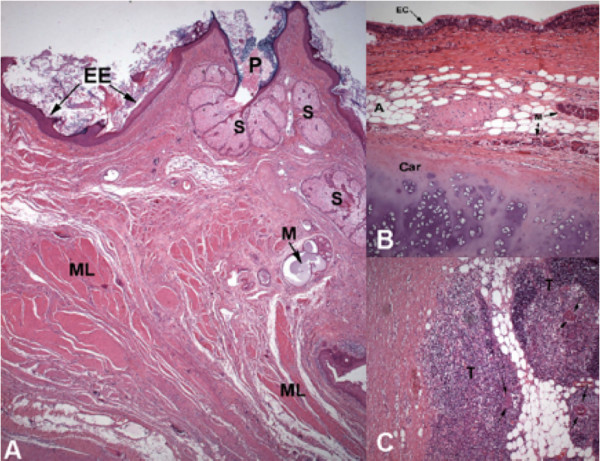
**Histological sections of mature cystic teratoma (hematoxylin and eosin stain)**. **(A) **Skin (SK), mucous (M), and sebaceous glands (S) and smooth muscle (SM) (lens objective, ×5). **(B) **Ciliated columnar epithelium (CCE), cartilage (Car), mucous glands (M), and adipose tissue (A) (lens objective, ×10). **(C) **Thymus (T) and Hassall's corpuscles (arrows) (lens objective, ×10).

Gross examination showed a rounded tumor measuring 8.0cm × 8.0cm × 4.0cm and weighing 66g. The tumor was predominantly cystic, with a thin, sharply delineated wall filled with sebaceous material and hair. Microscopically, the cyst wall was lined by stratified squamous epithelium with underlying sebaceous glands and hair follicles or by simple ciliated columnar epithelium. Cartilage, adipose tissue, and smooth muscle were also seen in the cyst wall (Figures 3A and 3B). A histological diagnosis of a mature cystic teratoma was made once immature epithelial, mesenchymal, or neural elements were not found and there was no morphological evidence of malignancy in the tumor. Atrophic thymus was found in the tissue that surrounded the tumor (Figure 3C). The patient had a good post-operative recovery and was discharged to home on the sixth post-operative day.

## Discussion

This case report illustrates the accuracy of complementary examinations. All the investigations suggested a cystic tumor, probably of pericardial origin. Sometimes it is hard to diagnose a teratoma on the basis of imaging examinations. To make the diagnosis of teratoma, it is mandatory to find at least two of three germ layers [2,3]. The ectoderm tissue generally is predominant and is composed of neural tissues, skin, hair, and teeth. Mesodermic tissues such as fat, cartilage, or bone and endodermic tissue are less common. The endoderm layer is characterized by respiratory or intestinal epithelia. The complementary examinations' diagnostic accuracy is high if the characteristics of fat and teeth are identified in the tumor at the same time. The presence of just calcification, as in this case report, is not sufficient to characterize a teratoma, because calcification can also be found in tumors other than germ cell tumors [2,3].

## Conclusion

We relate an original report of a mature teratoma causing obliteration of the right cardiophrenic sinus with extrinsic heart compression. The correct diagnosis was made by using a surgical approach.

## Consent

Written informed consent was obtained from the patient for publication of this case report and any accompanying images. A copy of the written consent is available for review by the Editor-in-Chief of this journal.

## Competing interests

The authors declare that they have no competing interests.

## Authors' contributions

RV carried out the medical management and wrote the case report. HG carried out the post-operative management and wrote the case report. KU carried out the acquisition and analysis of the computed tomographic scan. LD participated in the histological diagnosis. JT was involved in the acquisition of echocardiographic images. NL carried out the post-operative management and helped to draft the manuscript. WH conceived of, coordinated, and participated in the elaboration of the case report. All authors read and approved the final manuscript.
